# Health Outcomes Among Long-term Opioid Users With Testosterone Prescription in the Veterans Health Administration

**DOI:** 10.1001/jamanetworkopen.2019.17141

**Published:** 2019-12-11

**Authors:** Guneet K. Jasuja, Omid Ameli, Joel I. Reisman, Adam J. Rose, Donald R. Miller, Dan R. Berlowitz, Shalender Bhasin

**Affiliations:** 1 Center for Healthcare Organization and Implementation Research, Edith Nourse Rogers Memorial Veterans Hospital, Veterans Administration Medical Center, Bedford, Massachusetts; 2 Department of Health Law, Policy and Management, Boston University School of Public Health, Boston, Massachusetts; 3 OptumLabs, Cambridge, Massachusetts; 4 Section of General Internal Medicine, Department of Medicine, Boston University School of Medicine, Boston, Massachusetts; 5 Research Program in Men’s Health, Aging and Metabolism, Boston Claude D. Pepper Older Americans Independence Center, Brigham and Women’s Hospital, Harvard Medical School, Boston, Massachusetts

## Abstract

**Importance:**

Androgen deficiency is common among male opioid users, and opioid use has emerged as a common antecedent of testosterone treatment. The long-term health outcomes associated with testosterone therapy remain unknown, however.

**Objective:**

To compare health outcomes between long-term opioid users with testosterone deficiency who filled testosterone prescriptions and those with the same condition but who did not receive testosterone treatment.

**Design, Setting, and Participants:**

This cohort study focused on men in the care of the Veterans Health Administration (VHA) facilities throughout the United States from October 1, 2008, to September 30, 2014. It included male veterans who were long-term opioid users, had low testosterone levels (<300 ng/dL), and received either a testosterone prescription or any other prescription. It excluded male patients with HIV infection, gender dysphoria, or prostate cancer and those who received testosterone in fiscal year 2008. Data were analyzed from April 1, 2017, to April 30, 2019.

**Exposure:**

Prescription for testosterone.

**Main Outcomes and Measures:**

All-cause mortality and incidence of major adverse cardiovascular events (MACE), vertebral or femoral fractures, and anemia during the 6-year follow-up through September 30, 2015.

**Results:**

After exclusions, 21 272 long-term opioid users (mean [SD] age, 53 [10] years; n = 16 689 [78.5%] white) with low total or free testosterone levels were included for analysis, of whom 14 121 (66.4%) received testosterone and 7151 (33.6%) did not. At baseline, compared with opioid users who did not receive testosterone, long-term opioid users who received testosterone treatment were more likely to have obesity (43.7% vs 49.0%; *P* < .001), hyperlipidemia (43.0% vs 48.8%; *P* < .001), and hypertension (53.9% vs 55.2%; *P* = .07) but had lower prevalence of coronary artery disease (15.9% vs 12.9%; *P* < .001) and stroke (2.4% vs 1.3%; *P* < .001). After adjusting for covariates, opioid users who received testosterone had significantly lower all-cause mortality (hazard ratio [HR] = 0.51; 95% CI, 0.42-0.61) and lower incidence of MACE (HR = 0.58; 95% CI, 0.51-0.67), femoral or hip fractures (HR = 0.68; 95% CI, 0.48-0.96), and anemia (HR = 0.73; 95% CI, 0.68-0.79) during the follow-up period of up to 6 years, compared with their counterparts without a testosterone prescription. In covariate-adjusted models, men who received opioids plus testosterone were more likely to have resolved anemia compared with those who received opioids only during the 6-year follow-up (HR = 1.16; 95% CI, 1.02-1.31). Similar results were obtained in propensity score–matched models and when analyses were restricted to opioid users with noncancer pain or those who did not receive glucocorticoids.

**Conclusions and Relevance:**

This study found that, in the VHA system, male long-term opioid users with testosterone deficiency who were treated with opioid and testosterone medications had significantly lower all-cause mortality and significantly lower incidence of MACE, femoral or hip fractures, and anemia after a multiyear follow-up. These results warrant confirmation through a randomized clinical trial to ascertain the efficacy of testosterone in improving health outcomes for opioid users with androgen deficiency.

## Key Points

**Question:**

What are the health outcomes among long-term opioid users who receive testosterone treatment compared with opioid users who do not?

**Findings:**

In this cohort study of 21 272 male long-term opioid users with testosterone deficiency, those who received opioids plus testosterone therapy had significantly lower all-cause mortality and lower incidence of major adverse cardiovascular events, anemia, and femoral or hip fractures than their counterparts who received opioids only in covariate-adjusted and propensity score–matched models.

**Meaning:**

This study’s findings suggest that receiving opioids plus testosterone treatment is associated with lower all-cause mortality and a lower incidence of other adverse health outcomes among men with opioid-induced androgen deficiency.

## Teaser

This cohort study analyzes the association between testosterone and opioid use and improved health outcomes among male US military veterans with opioid-induced androgen deficiency.

## Introduction

Opioid use in the United States has reached epidemic proportions.^1^ Although deaths from opioid overdose^2,3^ have captured the world’s attention, the endocrine complications of chronic opioid use have remained underappreciated. All opioid medications, especially long-acting opioids, suppress testosterone levels, often into the severely hypogonadal range.^4,5^ Prescription opioid use has been shown to have an association with testosterone deficiency; nearly 20% of testosterone prescriptions written in the Veterans Health Administration (VHA) system were for men who were opioid users.^6^ Testosterone deficiency among opioid users is associated with increased risk of sexual dysfunction, osteoporosis, and bone fractures.^7^ However, to our knowledge, no data exist on major health outcomes in opioid users who are hypogonadal and either receive or do not receive testosterone treatment.

Testosterone treatment of men with hypogonadism is associated with improved sexual desire, erections, and sexual activity^8,9^; self-reported mobility^10,11,12^; volumetric bone density and estimated bone strength^13,14^; and corrected anemia.^15^ However, the long-term association of testosterone treatment with major adverse cardiovascular events (MACE), mortality, and bone fractures remains unknown.^16,17,18,19,20,21,22,23^ Little is known about the effects of testosterone treatment on health outcomes in men with opioid-induced androgen deficiency (OPIAD), who often experience marked suppression of testosterone levels. One short-term randomized clinical trial reported improvements in pain sensitivity, sexual desire, and body composition with testosterone treatment in men with OPIAD.^24^ However, the long-term implications of testosterone treatment for major health outcomes in opioid users are unknown to date. Patients who receive opioids experience more severe testosterone deficiency, often have multiple comorbid conditions, use multiple prescription drugs, and are at increased risk of mortality,^25^ which could influence the benefit to risk ratio of testosterone treatment.

In the absence of a long-term randomized clinical trial of testosterone in men with OPIAD, we compared major health outcomes in male long-term opioid users who received testosterone treatment with those who did not receive testosterone. We selected health outcomes, including overall mortality, MACE, fractures (vertebral, femoral, and hip), and incident anemia, that have public health importance and could be ascertained with accuracy. Most randomized testosterone trials^14^ have been conducted in men with mild testosterone deficiency. Because opioid use is typically associated with moderate to severe testosterone deficiency, a study of men with OPIAD offers an opportunity to elucidate the association between testosterone treatment and health outcomes among opioid users with moderate to severe testosterone deficiency. Recognizing the limitations of observational studies, we performed propensity matching and several sensitivity analyses to assess the potential effect of confounding owing to observed and unobserved differences between groups.

## Methods

### Study Design and Participants

This nationwide cohort study included men who received prescriptions within the VHA system from October 1, 2008, to September 30, 2014, and were followed up through September 30, 2015. The institutional review board of Bedford VHA Medical Center approved the study and waived the need for informed consent because deidentified data were used. This study followed the Strengthening the Reporting of Observational Studies in Epidemiology (STROBE) reporting guideline.

For primary analyses, we selected male veterans who were long-term opioid users, had testosterone deficiency, and received either a testosterone prescription (testosterone recipients) or any other prescription (nonrecipients of testosterone) in each of the 2 or more years after filling an opioid prescription. Opioids and their morphine equivalents are listed in eTable 1 in the Supplement. We focused on long-term opioid users, defined as those who received 120 or more days’ supply of opioids during at least 1 continuous 180-day interval^26^ in 2 or more years as indicated by prescription fills. To identify opioid users with testosterone deficiency, we selected those with testosterone levels measured before they received a testosterone prescription. Those with a low total testosterone level (<300 ng/dL; to convert to nanomoles per liter, multiply by 0.0347) or free testosterone level (<70 pg/mL) were included. These cutoff points for testosterone levels were consistent with thresholds in guidelines available at that time and with published reference ranges.^27,28^ We excluded, among others, those with HIV infection, gender dysphoria, and prostate cancer and those who received testosterone in fiscal year (FY) 2008 because these individuals could have been using testosterone before receiving an opioid prescription.

### Exposure and Outcome Intervals

For patients who received testosterone prescriptions, the earliest testosterone prescription fill between FY 2009 and 2014 was used as the index fill. For patients who did not receive testosterone prescriptions, a fill from FY 2009 to 2012 was chosen at random as the index fill in order to match the time distribution of testosterone fills.

To avoid the immortal person time bias,^29^ the exposure interval for testosterone recipients was calculated as the interval between the date of their first testosterone prescription and a date 3 months after their last testosterone fill. For nonrecipients of testosterone, the duration of exposure was calculated as the interval between the date of their most recent testosterone-level check preceding their index date and a date 3 months after their last opioid prescription. This definition of exposure period ensured that patients were actively in care at least at the start and end of the period and had sufficient care within the VHA system to be included in the present study cohort. The outcome period started at the beginning of the specific exposure period for both recipients and nonrecipients of testosterone.

### Outcomes and Covariates

We selected all-cause mortality and first occurrence of MACE as the primary outcomes of the study and vertebral, femoral, or hip fractures and anemia as the secondary outcomes. These outcomes were selected because they are clinically important and can be ascertained with a high level of accuracy. The date of death was extracted from the VHA Vital Status File, which is compiled by merging data from multiple sources to create a single date of death.^30^ Major adverse cardiovascular events included myocardial infarction, ischemic stroke, or death (defined with the *International Classification of Diseases, Ninth Revision, Clinical Modification,* [*ICD-9-CM*] codes [eTable 2 in the Supplement]). Vertebral, femoral, or hip fractures were identified using *ICD-9-CM* diagnosis codes in both outpatient and inpatient data from the VHA Corporate Data Warehouse (CDW) (eTable 2 in the Supplement).

Anemia was defined as hemoglobin level less than 12 g/dL (to convert to grams per liter, multiply by 10.0) or a hematocrit reading less than 36%^31^; the measurement closest to the index fill was used as the baseline level.

For anemia analyses, patients were required to have both baseline and follow-up hemoglobin or hematocrit values. We separately analyzed the resolution of anemia among those who had anemia at baseline (n = 1567) and the development of anemia among those who did not have anemia at baseline (n = 17 355).

We adjusted covariate-adjusted and propensity score–matched models for sociodemographic variables, including age, marital status, copayment, race/ethnicity, and poverty level in residential zip code. We also adjusted for a number of physical and mental comorbidities and specific medications (eTable 1 in the Supplement) using data from the VHA CDW. A 1-year look-back period was used to check for comorbidities and medications that occurred before the date of index prescription for testosterone recipients and nonrecipients of testosterone. These conditions were ascertained by the presence of at least 2 *ICD-9-CM* codes separated by 7 or more days (eTable 2 in the Supplement).

### Statistical Analysis

Baseline characteristics of study population and covariates were noted. We generated unadjusted bivariate- and covariate-adjusted Cox proportional hazards and propensity score–matched models. To minimize covariate imbalance between groups, we used a one-to-one propensity score match with a caliper of 0.001. Propensity scores were estimated using a logistic model, including demographic characteristics; coronary artery disease; hypertension; diabetes; hyperlipidemia; heart failure; stroke; chronic kidney disease; cancers; liver disease; dementia; depression; bipolar disease; indications for pain medication; substance use disorder; alcohol dependence; psychosis; and use of glucocorticoid, antidepressant, and antipsychotic medications.

We ran Cox proportional hazard models for up to 6 years of follow-up (FY 2009-2014). Testosterone recipients entered the analyses on the day of their index prescription fill and were followed up until 3 months after the date of their last testosterone fill. Nonrecipients of testosterone entered the analyses at the first date of their testosterone-level check and were followed up until 3 months after the date of their last opioid prescription. Study participants were censored at the earliest of the follow-up date, death, latest date of VHA service use, or September 30, 2015. Baseline comparisons between the recipients and nonrecipients of testosterone were performed with χ^2^ test, test of proportions, or unpaired, 2-tailed *t* test as appropriate. From the Cox models we determined hazard ratios (HRs) and 95% CIs associated with the testosterone recipients compared with the nonrecipients of testosterone. For models of outcomes, goodness of fit and proportional hazards assumptions were evaluated. We also used Kaplan-Meier curves to compare the unadjusted probability of survival separately for each primary outcome.

All analyses were conducted with SAS, version 9.4 (SAS Institute Inc). A statistical significance threshold of a 2-sided *P* = .05 was used. Data were analyzed from April 1, 2017, to April 30, 2019.

### Sensitivity Analyses

Because of the lack of randomization, confounding owing to baseline between-group differences was a potential challenge. Therefore, we conducted several sensitivity analyses to assess the robustness of associations with outcomes.

The first sensitivity analysis excluded patients who had a diagnosis of cancer (n = 20 366), given that the treatment of cancer pain can differ from that of noncancer pain.^32^ The second sensitivity analysis excluded men who received glucocorticoids (n = 15 149), given that these medications can suppress testosterone levels and affect outcomes.^33^ The third sensitivity analysis examined all-cause mortality and MACE outcomes as a function of different testosterone formulations (injections, gels, and patches), comparing testosterone recipients with nonrecipients because difference in outcomes have been reported with different formulations.^34^

The fourth sensitivity analysis used a simulation algorithm to examine the role of a potential unobserved confounder as discussed by Higashi et al.^35^ We assumed the omitted confounder to be a binary variable and simulated it to correlate with death in patients who received long-term opioid plus testosterone therapy. We estimated the degree of correlation that would be needed between the unmeasured confounder and outcome (death) and the exposure (use of opioid plus testosterone treatment) to explain the association with survival.

## Results

### Sample Characteristics

**Figure 1.**
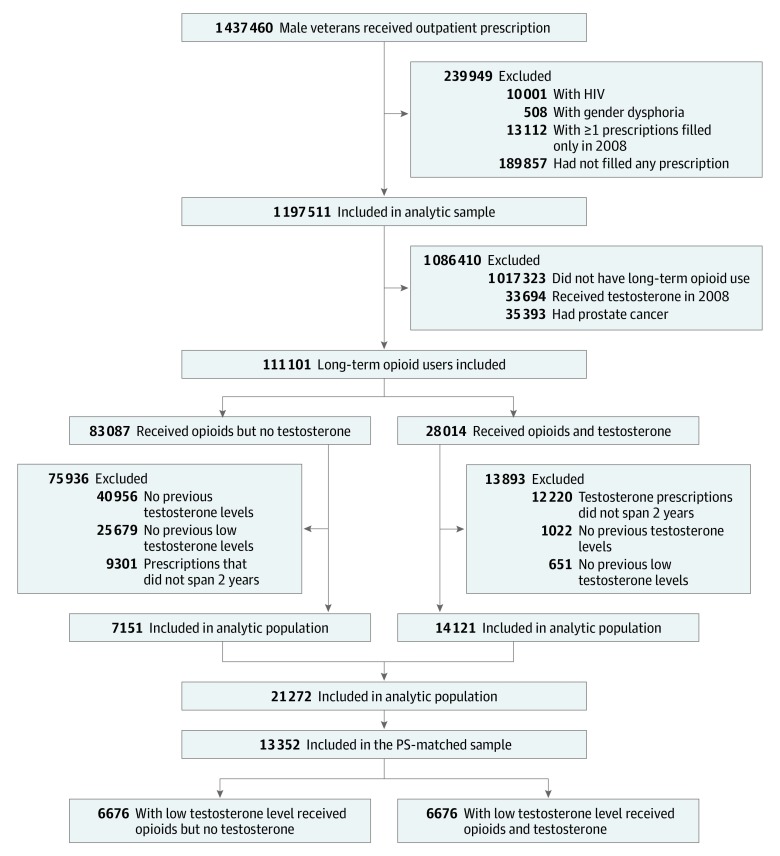
STROBE Diagram of Analytical Sample Selection Long-term opioid use was defined as use by patients who received 120 or more days’ supply of opioids during at least 1 continuous 180-day interval in 2 or more years. Low testosterone level was defined as total testosterone level of less than 300 ng/dL (to convert to nanomoles per liter, multiply by 0.0347) or free testosterone level of less than 70 pg/mL in the past 1 year. PS indicates propensity score.

Among the 1 437 460 men who received an outpatient prescription in a VHA facility between FY 2008 and FY 2014, we excluded 10 001 men (0.7%) with HIV infection, 508 (0.04%) with gender dysphoria, 13 112 (0.9%) with 1 or more prescriptions filled only in FY 2008 but not before, and 189 857 (13.2%) without any filled prescription in FY 2008 (Figure 1). We excluded 33 694 men (2.3%) who received testosterone in FY 2008; 35 393 (2.5%) who had prostate cancer; and 1 017 323 (70.8%) who received no opioids, received opioids but less than the 120 days’ supply during any continuous 180 days, or received opioids but for less than 1 year from earliest to latest opioid prescription. From the 111 101 long-term opioid users, we selected those who received testosterone prescription fills for 2 or more years (testosterone recipients) or any other prescription fill for 2 or more years (nonrecipients of testosterone). In all, 21 272 men (19.1%) with a total testosterone level less than 300 ng/dL or free testosterone level less than 70 pg/mL were included in the analyses.

Among the 21 272 long-term opioid users in this study, 14 121 (66.4%) received testosterone and 7151 (33.6%) did not. The race/ethnicity of most patients was white (n = 16 689 [78.5%]), and the mean (SD) age of the entire sample was 53 (10) years (Table 1). Compared with opioid users who did not receive testosterone, a slightly higher proportion of opioid users who received testosterone had hypertension (53.9% vs 55.2%; *P* = .07), hyperlipidemia (43.0% vs 48.8%; *P* < .001), obesity (43.7% vs 49.0%; *P* < .001), and posttraumatic stress disorder (24.2% vs 25.6%; *P* = .02) and were more likely to be receiving an opioid dose greater than 50 morphine milligram equivalents (MME) (29.4% vs 42.8%; *P* < .001). A slightly lower proportion of testosterone recipients compared with nonrecipients had prevalent coronary artery disease (12.9% vs 15.9%; *P* < .001) and stroke (1.3% vs 2.4%; *P* < .001). The distribution of these characteristics in the 2 groups was similar after the propensity score match, as expected.

**Table 1.** Baseline Characteristics of the Sample

Variable	Before Propensity Matching, %^a^		After Propensity Matching, %^a^	
	Nonrecipients of Testosterone	Recipients of Testosterone	Nonrecipients of Testosterone	Recipients of Testosterone
No. of participants	7151	14 121	6676	6676
Age, mean (SD), y	54.8 (10.6)^b^	52.8 (10.0)^b^	54.3 (10.5)	54.2 (10.5)
Age, y				
20-39	8.9^b^	10.6^b^	9.3	9.6
40-49	17.1^b^	21.2^b^	17.9	17.8
50-59	42.5^b^	43.9^b^	42.8	43.0
60-69	24.6^b^	21.1^b^	24.4	24.1
70-79	5.6^b^	2.7^b^	4.6	4.6
≥80	1.2^b^	0.5^b^	0.9	0.9
Race/ethnicity				
Non-Hispanic				
White	74.4^b^	80.5^b^	76.0	76.1
Black	13.8^b^	8.4^b^	12.3	12.3
Hispanic	4.1^b^	3.4^b^	4.0	4.1
Other, specified	2.3^b^	2.3^b^	2.3	2.2
Unknown	5.4^b^	5.5^b^	5.4	5.3
BMI, mean (SD)	31.3 (6.4)^b^	32.6 (6.7)^b^	31.6 (6.4)	31.8 (6.6)
BMI				
≤18.4	0.5^b^	0.4^b^	0.4	0.4
18.5-24 (Normal weight)	13.6^b^	9.0^b^	12.4	12.4
25-29 (Overweight)	32.6^b^	29.4^b^	32.0	32.0
30-39 (Obesity)	43.7^b^	49.0^b^	45.1	44.8
≥40 (Morbid obesity)	9.6^b^	12.3^b^	10.0	10.3
Diabetes	35.6^b^	32.7^b^	35.2	35.1
Hypertension	53.9	55.2	53.8	53.2
Hyperlipidemia	43.0^b^	48.8^b^	44.1	43.2
COPD	15.3^c^	13.7^c^	15.0	14.8
Obstructive sleep apnea	5.9	5.3	5.8	5.7
CHF	5.1 ^b^	3.3 ^b^	4.5	4.5
Coronary artery disease	15.9 ^b^	12.9 ^b^	15.1	15.0
Stroke	2.4 ^b^	1.3 ^b^	2.0	1.9
TIA	0.3	0.4	0.4	0.3
Peripheral artery disease	5.4 ^b^	3.1 ^b^	4.7	4.1
Chronic kidney disease	5.3^b^	3.2^b^	4.6	4.5
Bipolar disorder	5.4^c^	6.6^c^	5.6	5.8
Antidepressant use	61.5^b^	69.6^b^	63.2	63.5
Anxiety disorder	11.8^b^	13.9^b^	12.1	12.4
PTSD	24.2^c^	25.6^c^	24.6	24.6
Alcohol abuse	6.8	6.2	6.6	6.5
Glucocorticoid use, systemic	29.1	28.6	28.9	29.2
Baseline total testosterone, mean (SD), ng/dL	248.7 (88.1)^b^	174.3 (81.2)^b^	248.6 (87.5)^b^	178.3 (81.4)^b^
Formulation of testosterone				
Injection	NA	62.7	NA	62.3
Gel	NA	14.6	NA	14.5
Patch	NA	22.7	NA	23.2
High-dose opioid therapy >50 MME	29.4^b^	42.8^b^	31.1	31.1
Exposure time, d				
Mean (SD)	654 (470)^b^	1034 (447)^b^	654 (470)^b^	1012 (442)^b^
Median (range)	752 (366-2280)	929 (369-2274)	754 (366-2280)	900 (370-2274)

Abbreviations: BMI, body mass index (calculated as weight in kilograms divided by height in meters squared); CHF, congestive heart failure; COPD, chronic obstructive pulmonary disease; MME, morphine milligram equivalents; NA, not applicable; PTSD, posttraumatic stress disorder; TIA, transient ischemic attack.

SI conversion factor: To convert testosterone from nanograms per deciliter to nanomoles per liter, multiply by 0.0347.

^a^ Results of statistical comparison between those who received testosterone and those who did not. *P* > .05 unless otherwise indicated.

^b^ *P* < .001

^c^ Between *P* = .001 and *P* = .05.

### All-Cause Mortality, MACE, Fractures, and Anemia

**Figure 2.**
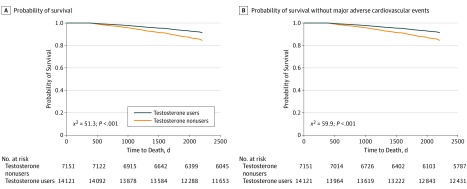
Kaplan-Meier Curves of Survival Probability Overall and Without Major Adverse Cardiovascular Events During 6-Year Follow-up Y-axes show covariate-adjusted survival rates. Orange line indicates long-term opioid users who did not receive testosterone; navy line, long-term opioid users who received testosterone treatment.

In unadjusted and covariate-adjusted models, men who received opioid plus testosterone therapy had statistically significantly lower all-cause mortality than men who received opioids only during the follow-up period of up to 6 years (HR = 0.51; 95% CI, 0.42-0.61) (Table 2 and Figure 2). The incidence of MACE was also significantly lower in recipients of opioids plus testosterone treatment compared with recipients of opioids only during follow-up in the covariate-adjusted models (HR = 0.58; 95% CI, 0.51-0.67).

**Table 2.** Likelihood of Outcomes in the 6-Year Follow-up Period as a Function of Long-term Opioid or Testosterone Use Status

Outcome	Unadjusted Estimates		HR (95% CI)	
	Outcome Events, No. (Unadjusted Incidence Rate per 100 Person-Years)	Bivariate HR (95% CI)	Covariate-Adjusted Cox Model (Model 1)^a^	PS-Matched Cox Model (Model 2)
No.	NA	21 272	21 272	13 352
All-cause mortality				
No testosterone	203 (1.4)	1 [Reference]	1 [Reference]	1 [Reference]
Testosterone	327 (0.7)	0.41 (0.34-0.49)	0.51 (0.42-0.61)	0.54 (0.44-0.67)
Incidence of MACE or deaths^b^				
No testosterone	358 (2.5)	1 [Reference]	1 [Reference]	1 [Reference]
Testosterone	605 (1.2)	0.48 (0.42-0.54)	0.58 (0.51-0.67)	0.60 (0.52-0.70)
Incidence of vertebral fractures (*ICD-9-CM* codes 805 and 806)				
No testosterone	60 (0.43)	1 [Reference]	1 [Reference]	1 [Reference]
Testosterone	154 (0.31)	0.80 (0.59-1.08)	0.86 (0.63-1.18)	0.91 (0.64-1.30)
Incidence of femoral or hip fractures (*ICD-9-CM* codes 808, 820, and 821)				
No testosterone	55 (0.39)	1 [Reference]	1 [Reference]	1 [Reference]
Testosterone	94 (0.19)	0.54 (0.39-0.76)	0.68 (0.48-0.96)	0.60 (0.40-0.89)
Incidence of vertebral, femoral, or hip fractures (*ICD-9-CM* codes 805, 806, 808, 820, and 821)				
No testosterone	107 (0.76)	1 [Reference]	1 [Reference]	1 [Reference]
Testosterone	239 (0.48)	0.70 (0.59-0.88)	0.80 (0.63-1.01)	0.78 (0.59-1.02)
Anemia				
Subgroup 1: patients with baseline anemia^c^				
Baseline anemia resolved, No.	NA	1567	1567	876
No testosterone	469 (115.6)	1 [Reference]	1 [Reference]	1 [Reference]
Testosterone	837 (133.2)	1.18 (1.06-1.33)	1.16 (1.02-1.31)	1.17 (1.01-1.35)
Subgroup 2: patients without baseline anemia^c^				
New anemia emerged, No.	NA	17 355	17 355	10 596
No testosterone	1104 (13.0)	1 [Reference]	1 [Reference]	1 [Reference]
Testosterone	2205 (7.6)	0.63 (0.59-0.68)	0.73 (0.68-0.79)	0.73 (0.67-0.80)

Abbreviations: HR, hazard ratio; *ICD-9-CM, International Classification of Diseases, Ninth Revision, Clinical Modification*; MACE, major adverse cardiovascular events; NA, not applicable; PS, propensity score.

^a^ Adjusted for age, race/ethnicity, marital status, body mass index, copay requirement, zip code poverty level, and baseline status of the following clinical conditions: indications for pain, chronic pain conditions, use of glucocorticoid medications, congestive heart failure, cancers, coronary artery disease, hypertension, diabetes, hyperlipidemia, liver disease, chronic kidney disease, stroke or transient ischemic attack, dementia, depression, bipolar disease, substance use disorder, alcohol dependence, psychosis, and use of antipsychotic medications.

^b^ Incident cases (new occurrence) of myocardial infarction or thrombotic stroke or death (eTable 2 in the Supplement).

^c^ Based on measurements closest to the index date. Anemia is defined as hemoglobin level less than 12 g/dL (to convert to grams per liter, multiply by 10.0) or a hematocrit reading less than 36%. Included patients had to have both preindex and postindex hemoglobin or hematocrit values.

The incidence of femoral and hip fractures was significantly lower in recipients of opioids plus testosterone than in recipients of opioids only in unadjusted and covariate-adjusted models during follow-up (HR = 0.68; 95% CI, 0.48-0.96). When vertebral fractures were considered individually (HR = 0.86; 95% CI, 0.63-1.18) or when all fractures (vertebral plus femoral and hip fractures) were considered (HR = 0.80; 95% CI, 0.63-1.01), the associations with testosterone treatment were not significant.

Among participants who were anemic at baseline, testosterone treatment was significantly associated with the resolution of anemia during the 6-year follow-up (HR = 1.16; 95% CI, 1.02-1.31) in the covariate-adjusted model. Long-term opioid users who received testosterone had a significantly lower risk of incident anemia compared with opioid users who did not receive testosterone (HR = 0.73; 95% CI, 0.68-0.79). The findings of propensity score–adjusted models were similar to those of the covariate-adjusted models (Table 2). Cox proportional hazards models for propensity score–matched samples demonstrated a significantly lower hazard for all-cause mortality (HR = 0.54; 95% CI, 0.44-0.67); significantly lower incidence of MACE (HR = 0.60; 95% CI, 0.52-0.70), femoral or hip fractures (HR = 0.60; 95% CI, 0.40-0.89), and anemia (HR = 0.73; 95% CI, 0.67-0.80); and significantly higher rates of resolved anemia (HR = 1.17; 95% CI, 1.01-1.35) for testosterone recipients compared with nonrecipients.

### Sensitivity Analyses

In a sensitivity analysis that excluded men with cancer pain, testosterone recipients in covariate-adjusted models had significantly lower all-cause mortality (HR, 0.51; 95% CI, 0.42-0.62) and lower incidence of MACE (HR, 0.58; 95% CI, 0.50-0.67), femoral or hip fracture (HR, 0.65; 95% CI, 0.45-0.94), and anemia (HR, 0.74; 95% CI, 0.68-0.80) compared with nonrecipients of testosterone, a finding consistent with that of the primary analysis (Table 3). When the analysis was limited to patients who did not receive glucocorticoid medication, with the exception of femoral or hip fractures, the results were comparable (mortality: HR, 0.56 [95% CI, 0.44-0.71]; MACE: HR, 0.57 [95% CI, 0.48-0.68]; anemia: HR, 0.71 [95% CI, 0.64-0.78]) (eTable 3 in the Supplement).

**Table 3.** Subgroup (Patients Without Cancer) Analysis of Outcomes in the 6-Year Follow-up Period as a Function of Long-term Opioid or Testosterone Use Status

Outcome	Unadjusted Estimates		HR (95% CI)	
	Outcome Events, No. (Unadjusted Incidence Rate per 100 Person-Years)	Bivariate HR (95% CI)	Covariate-Adjusted Cox Model (Model 1)^a^	PS-Matched Cox Model (Model 2)
No.	NA	20 366	20 366	12 702
All-cause mortality				
No testosterone	179 (1.3)	1 [Reference]	1 [Reference]	1 [Reference]
Testosterone	298 (0.6)	0.42 (0.35-0.50)	0.51 (0.42-0.62)	0.53 (0.42-0.66)
Incidence of MACE or deaths^b^				
No testosterone	328 (2.4)	1 [Reference]	1 [Reference]	1 [Reference]
Testosterone	563 (1.2)	0.48 (0.42-0.55)	0.58 (0.50-0.67)	0.59 (0.50-0.69)
Incidence of vertebral fractures (*ICD-9-CM* codes 805 and 806)				
No testosterone	55 (0.41)	1 [Reference]	1 [Reference]	1 [Reference]
Testosterone	145 (0.30)	0.81 (0.59-1.11)	0.87 (0.63-1.20)	0.91 (0.63-1.32)
Incidence of femoral or hip fractures (*ICD-9-CM* codes 808, 820, and 821)				
No testosterone	51 (0.38)	1 [Reference]	1 [Reference]	1 [Reference]
Testosterone	85 (0.18)	0.52 (0.37-0.74)	0.65 (0.45-0.94)	0.56 (0.37-0.85)
Incidence of vertebral, femoral, or hip fractures (*ICD-9-CM* codes 805, 806, 808, 820, and 821)				
No testosterone	98 (0.73)	1 [Reference]	1 [Reference]	1 [Reference]
Testosterone	223 (0.47)	0.71 (0.56-0.90)	0.80 (0.62-1.03)	0.77 (0.58-1.03)
Anemia				
Subgroup 1: patients with baseline anemia^c^				
Baseline anemia resolved, No.	NA	1399	1399	782
No testosterone	399 (112.9)	1 [Reference]	1 [Reference]	1 [Reference]
Testosterone	771 (131.5)	1.19 (1.06-1.35)	1.14 (1.00-1.30)	1.17 (1.00-1.37)
Subgroup 2: patients without baseline anemia^c^				
New anemia emerged, No.	NA	16 667	16 667	10 150
No testosterone	1019 (12.5)	1 [Reference]	1 [Reference]	1 [Reference]
Testosterone	2067 (7.4)	0.64 (0.59-0.69)	0.74 (0.68-0.80)	0.73 (0.67-0.80)

Abbreviations: HR, hazard ratio; *ICD-9-CM, International Classification of Diseases, Ninth Revision, Clinical Modification*; MACE, major adverse cardiovascular events; NA, not applicable; PS, propensity score.

^a^ See note a in Table 2.

^b^ See note b in Table 2.

^c^ See note c in Table 2.

In sensitivity analyses comparing different testosterone formulations on all-cause mortality and MACE, all testosterone formulations were associated with lower likelihood of mortality (HR, 0.51; 95% CI, 0.42-0.61) and MACE (HR, 0.58; 95% CI, 0.51-0.67) in fully adjusted models comparing testosterone recipients with nonrecipients (eTable 4 in the Supplement).

We examined the potential role of an unobserved confounder and identified the critical levels for correlation of a potential confounder with all-cause mortality and testosterone exposure that would completely explain the observed effects (eTable 5 in the Supplement). The association between treatment with opioid plus testosterone therapy and mortality was moderately sensitive to unmeasured confounding. An unmeasured confounder with 0.3 correlation with opioid plus testosterone treatment would need to have a −0.06 (HR, 1.09; 95% CI, 0.90-1.32; *P* = .37) correlation or stronger with mortality to eliminate the negative implication of testosterone for mortality.

## Discussion

Among long-term opioid users in the VHA, men who received opioid plus testosterone therapy had significantly lower all-cause mortality and significantly lower incidence of MACE, anemia, and femoral or hip fractures compared with men who received opioid treatment alone. The association between testosterone treatment and MACE and mortality was robust to analyses, which used both covariate-adjusted and propensity score–matched models. Sensitivity analyses in men who had noncancer pain or who did not receive glucocorticoids confirmed the findings of the primary analysis. This cohort study, a first step toward understanding the association of testosterone treatment with health outcomes in long-term opioid users, has clinical implications because of the high prevalence of opioid use among US military veterans and high rates of androgen deficiency and testosterone use among opioid users. These findings need confirmation in a randomized clinical trial.

Because assignment to testosterone treatment was not randomized, the differences in outcomes between those treated with opioids plus testosterone and those who received opioids only cannot be attributed with certainty to testosterone treatment. Although differences in outcomes remained statistically significant even after propensity score matching, baseline differences between patient groups may have been factors in outcomes in ways not captured by propensity score. Compared with men who received opioids only, men who received opioids plus testosterone had a higher mean body mass index and higher prevalence of hyperlipidemia, hypertension, and psychiatric disorders. The men who received opioids only had slightly higher prevalence of some other comorbid conditions, including coronary artery disease and stroke. We attempted to address this concern by propensity score matching and by simulating the implication of a potential unobserved confounder. The simulation analyses showed that an unobserved confounder (eg, better access to health care) would need to be moderately correlated with both the exposure (receipt of testosterone and opioids) and the outcome (mortality) to explain these findings beyond adjustment for observed covariates.

Because we used propensity score matching to account for baseline differences between groups, the existence of such strong, unmeasured confounding was not likely, but it cannot be ruled out entirely. Propensity scores can balance only observed covariates, and findings could be subject to bias from unmeasured confounding variables. Furthermore, establishing the index date for individuals not receiving medications is complex. On the basis of similar previous approaches to identifying an index date for individuals not receiving medications,^36,37^ we used the date of documented testosterone level as the beginning of exposure for nonrecipients of testosterone.

To our knowledge, this cohort study is the first to examine the association between testosterone treatment in long-term opioid users and MACE, fractures, anemia, and all-cause mortality. The only short-term randomized clinical trial in men with OPIAD found that testosterone treatment improved sexual function, pain sensitivity, and body composition.^24^ Similar improvements in sexual function^38^ have been reported in uncontrolled short-term studies in patients treated with opioid medications.^39^ Opioid agonists used in the treatment of opioid addiction and treatment have been shown to differently affect testosterone levels and sexual function in opioid-dependent men. Compared with methadone, buprenorphine has been found to be associated with substantially lower suppression of testosterone levels and with lower rates of sexual dysfunction.^40,41^

These findings are in contrast to reports that testosterone therapy was associated with adverse cardiovascular events in men with low testosterone, most of whom did not have long-term use of opioids.^16,17,18^ However, not all studies found this association between testosterone and increased risk of death or cardiovascular outcomes, and some studies even reported cardiovascular advantages.^19,20,42^

### Strengths and Limitations

This study has several strengths, one of which is its use of the large and detailed VHA CDW. The CDW included medication dispensing records, laboratory results, *ICD-9-CM* diagnosis codes, and demographics. The large sample size and long follow-up period enabled the reliable assessment of MACE, mortality, and fracture outcomes, which would not have been possible in smaller studies of shorter duration. The study included hard outcomes that are clinically important. The concern about confounding by indication owing to testosterone recipients having low testosterone levels at baseline was accounted for by requiring control patients to meet that criterion as well. In addition, we used propensity score matching to minimize the factors in baseline differences, and we performed additional sensitivity analyses to assess the effect of unmeasured confounding. We included only patients who received an opioid prescription in the year before receiving a testosterone prescription and who had a confirmed low total or free testosterone level before receiving testosterone. To avoid the immortal person time bias,^29^ we defined the exposure by the dates of the first and last testosterone prescription fills.

This study’s limitations include its observational design, whereby unmeasured confounding could have affected the findings. Physicians also could have selected healthier patients to receive testosterone therapy. We attempted to control for confounding by adjusting for a wide range of relevant demographics, conditions, and medications; by using propensity score matching; and by simulating the potential effect of an unobserved confounder. However, unmeasured confounding cannot be fully excluded. The quality of the assays for total and free testosterone levels within the VHA varied. Immunoassays for testosterone commonly used in this period were susceptible to inaccuracy in the low range; some patients on testosterone treatment might not have been hypogonadal, whereas some men with hypogonadism may have been misdiagnosed as eugonadal. The protective influence of testosterone on mortality in patients taking opioids remained robust in each of these different analyses. We only had access to VHA pharmacy data; some patients may have filled a testosterone prescription outside the system. Because medication costs in the VHA system were lower than outside the system, it was unlikely that many patients obtained their testosterone prescription externally. Furthermore, this factor would only dilute the treatment effect and drive the results toward null.

Sexual function, quality of life, and well-being outcomes could not be ascertained from the VHA CDW, and mental health outcomes were not assessed. Although *ICD-9-CM* codes are commonly used to identify conditions, these codes may not have been recorded accurately. Study outcomes were ascertained based on clinical coding and were not adjudicated. The total number of fractures was small, and the study may not have had sufficient statistical power to detect between-group differences in fracture events. Opioid use was defined according to prescription fills, and low testosterone levels were identified by documented laboratory results. On-treatment testosterone levels in men who received testosterone treatment varied widely^43^; however, testosterone levels during treatment were not consistently monitored. Although we required all patients to have documented opioid prescriptions in 2 or more years, a patient in either group could have stopped using opioids or could have changed their use pattern within the study period. Patients in the VHA system typically have a greater burden of comorbid conditions compared with the general population, which may affect the generalizability of these findings to non-VHA patients.^44^

## Conclusions

This cohort study found that, among men who were long-term opioid users, those who received a testosterone prescription had significantly lower all-cause mortality and a significantly lower incidence of MACE, anemia, and femoral or hip fractures in up to 6 years of follow-up compared with male opioid users who did not receive testosterone therapy. Because of the observational nature of this study, confounding owing to known and unknown differences between groups cannot be disregarded. Because of the high prevalence of opioid use among US veterans and the high proportion of opioid users who receive testosterone treatment, we believe a randomized clinical trial is warranted to ascertain whether testosterone treatment is safe and whether it is associated with improved health outcomes among opioid users who have androgen deficiency.

## Notes

Supplement. **eTable 1.** Medication Codes **eTable 2. **ICD-9-CM Diagnosis and Procedure Codes **eTable 3. **Subgroup Analysis of All-Cause Mortality, Major Adverse Cardiovascular Event, Bone Fractures, and Anemia Outcomes in the One-Year and the Six-Year Follow-up Time as a Function of Long-term Opioid/Testosterone Use Status: Patients Not on Glucocorticoids **eTable 4. **Analyses of Subsets of Testosterone Users Using a Specific Testosterone Formulation in Comparison With All Non-Testosterone Users **eTable 5. **Correlation of a Potentially Unobserved Confounder With All-Cause Mortality and Long-term Opioids With Testosterone

## References

[1] Guy GP Jr, Zhang K, Bohm MK, Vital signs: changes in opioid prescribing in the United States, 2006-2015. MMWR Morb Mortal Wkly Rep. 2017;66(26):-. doi:10.15585/mmwr.mm6626a4 28683056 PMC5726238

[2] Gomes T, Tadrous M, Mamdani MM, Paterson JM, Juurlink DN The burden of opioid-related mortality in the United States. JAMA Netw Open. 2018;1(2):e180217. doi:10.1001/jamanetworkopen.2018.0217 30646062 PMC6324425

[3] National Consensus Development Panel on Effective Medical Treatment of Opiate Addiction Effective medical treatment of opiate addiction. JAMA. 1998;280(22):1936-1943. doi:10.1001/jama.280.22.1936 9851480

[4] Cepeda MS, Zhu V, Vorsanger G, Eichenbaum G Effect of opioids on testosterone levels: cross-sectional study using NHANES. Pain Med. 2015;16(12):2235-2242. doi:10.1111/pme.12843 26177122

[5] Rubinstein AL, Carpenter DM, Minkoff JR Hypogonadism in men with chronic pain linked to the use of long-acting rather than short-acting opioids. Clin J Pain. 2013;29(10):840-845. doi:10.1097/AJP.0b013e31827c7b5d 24384986

[6] Jasuja GK, Bhasin S, Reisman JI, Who gets testosterone? patient characteristics associated with testosterone prescribing: a cross-sectional study. J Gen Intern Med. 2017;32(3):304-311. doi:10.1007/s11606-016-3940-7 27995426 PMC5331013

[7] Daniell HW Opioid-induced androgen deficiency discussion in opioid contracts. Am J Med. 2007;120(9):e21. doi:10.1016/j.amjmed.2006.05.027 17765033

[8] Cunningham GR, Stephens-Shields AJ, Rosen RC, Testosterone treatment and sexual function in older men with low testosterone levels. J Clin Endocrinol Metab. 2016;101(8):3096-3104. doi:10.1210/jc.2016-1645 27355400 PMC4971331

[9] Snyder PJ, Bhasin S, Cunningham GR, ; Testosterone Trials Investigators Effects of testosterone treatment in older men. N Engl J Med. 2016;374(7):611-624. doi:10.1056/NEJMoa1506119 26886521 PMC5209754

[10] Srinivas-Shankar U, Roberts SA, Connolly MJ, Effects of testosterone on muscle strength, physical function, body composition, and quality of life in intermediate-frail and frail elderly men: a randomized, double-blind, placebo-controlled study. J Clin Endocrinol Metab. 2010;95(2):639-650. doi:10.1210/jc.2009-1251 20061435

[11] Travison TG, Basaria S, Storer TW, Clinical meaningfulness of the changes in muscle performance and physical function associated with testosterone administration in older men with mobility limitation. J Gerontol A Biol Sci Med Sci. 2011;66(10):1090-1099. doi:10.1093/gerona/glr100 21697501 PMC3202898

[12] Storer TW, Basaria S, Traustadottir T, Effects of testosterone supplementation for 3 years on muscle performance and physical function in older men. J Clin Endocrinol Metab. 2017;102(2):583-593. 27754805 10.1210/jc.2016-2771 PMC5413164

[13] Snyder PJ, Kopperdahl DL, Stephens-Shields AJ, Effect of testosterone treatment on volumetric bone density and strength in older men with low testosterone: a controlled clinical trial. JAMA Intern Med. 2017;177(4):471-479. doi:10.1001/jamainternmed.2016.9539 28241231 PMC5433755

[14] Snyder PJ, Bhasin S, Cunningham GR, Lessons from the testosterone trials. Endocr Rev. 2018;39(3):369-386. doi:10.1210/er.2017-00234 29522088 PMC6287281

[15] Roy CN, Snyder PJ, Stephens-Shields AJ, Association of testosterone levels with anemia in older men: a controlled clinical trial. JAMA Intern Med. 2017;177(4):480-490. doi:10.1001/jamainternmed.2016.9540 28241237 PMC5433757

[16] Basaria S, Coviello AD, Travison TG, Adverse events associated with testosterone administration. N Engl J Med. 2010;363(2):109-122. doi:10.1056/NEJMoa1000485 20592293 PMC3440621

[17] Vigen R, O’Donnell CI, Barón AE, Association of testosterone therapy with mortality, myocardial infarction, and stroke in men with low testosterone levels. JAMA. 2013;310(17):1829-1836. doi:10.1001/jama.2013.280386 24193080

[18] Finkle WD, Greenland S, Ridgeway GK, Increased risk of non-fatal myocardial infarction following testosterone therapy prescription in men. PLoS One. 2014;9(1):e85805. doi:10.1371/journal.pone.0085805 24489673 PMC3905977

[19] Shores MM, Smith NL, Forsberg CW, Anawalt BD, Matsumoto AM Testosterone treatment and mortality in men with low testosterone levels. J Clin Endocrinol Metab. 2012;97(6):2050-2058. doi:10.1210/jc.2011-2591 22496507

[20] Sharma R, Oni OA, Gupta K, Normalization of testosterone level is associated with reduced incidence of myocardial infarction and mortality in men. Eur Heart J. 2015;36(40):2706-2715. doi:10.1093/eurheartj/ehv346 26248567

[21] Baillargeon J, Urban RJ, Kuo YF, Risk of myocardial infarction in older men receiving testosterone therapy. Ann Pharmacother. 2014;48(9):1138-1144. doi:10.1177/1060028014539918 24989174 PMC4282628

[22] Anderson JL, May HT, Lappé DL, Impact of testosterone replacement therapy on myocardial infarction, stroke, and death in men with low testosterone concentrations in an integrated health care system. Am J Cardiol. 2016;117(5):794-799. doi:10.1016/j.amjcard.2015.11.063 26772440

[23] Shores MM Testosterone treatment and cardiovascular events in prescription database studies. Asian J Androl. 2018;20(2):138-144. doi:10.4103/aja.aja_25_17 28816202 PMC5858096

[24] Basaria S, Travison TG, Alford D, Effects of testosterone replacement in men with opioid-induced androgen deficiency: a randomized controlled trial. Pain. 2015;156(2):280-288. doi:10.1097/01.j.pain.0000460308.86819.aa 25599449 PMC6036339

[25] Ray WA, Chung CP, Murray KT, Hall K, Stein CM Prescription of long-acting opioids and mortality in patients with chronic noncancer pain. JAMA. 2016;315(22):2415-2423. doi:10.1001/jama.2016.7789 27299617 PMC5030814

[26] Leider HL, Dhaliwal J, Davis EJ, Kulakodlu M, Buikema AR Healthcare costs and nonadherence among chronic opioid users. Am J Manag Care. 2011;17(1):32-40. 21348566

[27] Bhasin S, Brito JP, Cunningham GR, Testosterone therapy in men with hypogonadism: an Endocrine Society clinical practice guideline. J Clin Endocrinol Metab. 2018;103(5):1715-1744. doi:10.1210/jc.2018-00229 29562364

[28] Bhasin S, Pencina M, Jasuja GK, Reference ranges for testosterone in men generated using liquid chromatography tandem mass spectrometry in a community-based sample of healthy nonobese young men in the Framingham Heart Study and applied to three geographically distinct cohorts. J Clin Endocrinol Metab. 2011;96(8):2430-2439. doi:10.1210/jc.2010-3012 21697255 PMC3146796

[29] Suissa S Effectiveness of inhaled corticosteroids in chronic obstructive pulmonary disease: immortal time bias in observational studies. Am J Respir Crit Care Med. 2003;168(1):49-53. doi:10.1164/rccm.200210-1231OC 12663327

[30] Maynard C Ascertaining veterans’ vital status: VA data sources for mortality ascertainment and cause of death. VIREC Database and Methods Cyberseminar Series. https://www.hsrd.research.va.gov/for_researchers/cyber_seminars/archives/video_archive.cfm?SessionID=1242. Published March 6, 2017. Accessed May 15, 2018.

[31] Mayo Clinic Low hemoglobin count. https://www.mayoclinic.org/symptoms/low-hemoglobin/basics/definition/sym-20050760. Published April 7, 2018. Accessed January 30, 2019.

[32] Wiffen PJ, Wee B, Derry S, Bell RF, Moore RA Opioids for cancer pain - an overview of Cochrane reviews. Cochrane Database Syst Rev. 2017;7:CD012592. doi:10.1002/14651858.CD012592 28683172 PMC6483487

[33] MacAdams MR, White RH, Chipps BE Reduction of serum testosterone levels during chronic glucocorticoid therapy. Ann Intern Med. 1986;104(5):648-651. doi:10.7326/0003-4819-104-5-648 3083749

[34] Layton JB, Meier CR, Sharpless JL, Stürmer T, Jick SS, Brookhart MA Comparative safety of testosterone dosage forms [published correction appears in *JAMA Intern Med*. 2015;175(7):1248]. JAMA Intern Med. 2015;175(7):1187-1196. doi:10.1001/jamainternmed.2015.1573 25962056 PMC4494981

[35] Higashi T, Shekelle PG, Adams JL, Quality of care is associated with survival in vulnerable older patients. Ann Intern Med. 2005;143(4):274-281. doi:10.7326/0003-4819-143-4-200508160-00008 16103471

[36] Lund JL, Horváth-Puhó E, Komjáthiné Szépligeti S, Conditioning on future exposure to define study cohorts can induce bias: the case of low-dose acetylsalicylic acid and risk of major bleeding. Clin Epidemiol. 2017;9:611-626. doi:10.2147/CLEP.S147175 29200891 PMC5703173

[37] Schneeweiss S, Patrick AR, Stürmer T, Increasing levels of restriction in pharmacoepidemiologic database studies of elderly and comparison with randomized trial results. Med Care. 2007;45(10)(suppl 2):S131-S142. doi:10.1097/MLR.0b013e318070c08e 17909372 PMC2905666

[38] Daniell HW, Lentz R, Mazer NA Open-label pilot study of testosterone patch therapy in men with opioid-induced androgen deficiency. J Pain. 2006;7(3):200-210. doi:10.1016/j.jpain.2005.10.009 16516826

[39] Raheem OA, Patel SH, Sisul D, Furnish TJ, Hsieh TC The role of testosterone supplemental therapy in opioid-induced hypogonadism: a retrospective pilot analysis. Am J Mens Health. 2017;11(4):1208-1213. doi:10.1177/1557988316672396 28625114 PMC5675327

[40] Bliesener N, Albrecht S, Schwager A, Weckbecker K, Lichtermann D, Klingmüller D Plasma testosterone and sexual function in men receiving buprenorphine maintenance for opioid dependence. J Clin Endocrinol Metab. 2005;90(1):203-206. doi:10.1210/jc.2004-0929 15483091

[41] Hallinan R, Byrne A, Agho K, McMahon C, Tynan P, Attia J Erectile dysfunction in men receiving methadone and buprenorphine maintenance treatment. J Sex Med. 2008;5(3):684-692. doi:10.1111/j.1743-6109.2007.00702.x 18093096

[42] Oni OA, Dehkordi SHH, Jazayeri M-A, Relation of testosterone normalization to mortality and myocardial infarction in men with previous myocardial infarction. Am J Cardiol. 2019;124(8):1171-1178. doi:10.1016/j.amjcard.2019.07.019 31409450

[43] Bhasin S, Travison TG, O’Brien L, Contributors to the substantial variation in on-treatment testosterone levels in men receiving transdermal testosterone gels in randomized trials. Andrology. 2018;6(1):151-157. doi:10.1111/andr.12428 28981994

[44] Jasuja GK, Bhasin S, Reisman JI, Berlowitz DR, Rose AJ Ascertainment of testosterone prescribing practices in the VA. Med Care. 2015;53(9):746-752. doi:10.1097/MLR.0000000000000398 26196850

