# Supplementary material for: Health Outcomes Among Long-term Opioid Users With Testosterone Prescription in the Veterans Health Administration
Source: JAMA Netw Open. 2019 Dec 11;2(12):e1917141. doi: 10.1001/jamanetworkopen.2019.17141 (PMC6991198; doi:10.1001/jamanetworkopen.2019.17141)
Supplement: Supplement. — eTable 1. Medication Codes eTable 2. ICD-9-CM Diagnosis and Procedure Codes eTable 3. Subgroup Analysis of All-Cause Mortality, Major Adverse Cardiovascular Event, Bone Fractures, and Anemia Outcomes in the One-Year and the Six-Year Follow-up Time as a Function of Long-term Opioid/Testosterone Use Status: Patients Not on Glucocorticoids eTable 4. Analyses of Subsets of Testosterone Users Using a Specific Testosterone Formulation in Comparison With All Non-Testosterone Users eTable 5. Correlation of a Potentially Unobserved Confounder With All-Cause Mortality and Long-term Opioids With Testosterone [file jamanetwopen-2-e1917141-s001.pdf]

Supplementary Online Content

Jasuja GK, Ameli O, Reisman JI, et al. Health outcomes among long-term opioid users with testosterone prescription in the Veterans Health Administration. *JAMA Netw Open.* 2019;2(12):e1917141. doi:10.1001/jamanetworkopen.2019.17141

eTable 1. Medication Codes
eTable 2. ICD-9-CM Diagnosis and Procedure Codes
eTable 3. Subgroup Analysis of All-Cause Mortality, Major Adverse Cardiovascular Event, Bone Fractures, and Anemia Outcomes in the One-Year and the Six-Year Follow-up Time as a Function of Long-term Opioid/Testosterone Use Status: Patients Not on Glucocorticoids
eTable 4. Analyses of Subsets of Testosterone Users Using a Specific Testosterone Formulation in Comparison With All Non-Testosterone Users
eTable 5. Correlation of a Potentially Unobserved Confounder With All-Cause Mortality and Long-term Opioids With Testosterone

This supplementary material has been provided by the authors to give readers additional information about their work.

©2019 Jasuja GK et al. *JAMA Network Open.*

eTable 1. Medication Codes

	**Names/codes**	**Rule**
**Generic Names for Opioids**	Codeine (0.303)[a]<br>Dihydrocodeine (n/a)<br>Fentanyl (7.2)<br>Hydrocodone (1.0)<br>Hydromorphone (4.0)<br>Methadone (14.3)<br>Morphine (1.0)<br>Opium (1.0)<br>Oxycodone (1.5)	Used as part of the generic name text, stand alone or combination drug
**Antidepressants**	Amitriptyline, Amoxapine, Bupropion,Citalopram, Clomipramine, Desipramine, Desvenlafaxine, Doxepin, Duloxetine, Escitalopram, Fluoxetine, Fluvoxamine, Imipramine, Isocarboxazid, Maprotiline, Milnacipran, Mirtazapine, Nefazodone, Nortriptyline, Paroxetine, Phenelzine, Protriptyline, Selegiline, Sertraline, Tranylcypromine, Trazodone, Trimipramine, Venlafaxine, Vilazodone	Used as part of the generic name text, stand alone or combination drug
**Systemic Glucocorticoids**	Betamethasone<br>Budesonide<br>Cortisone<br>Dexamethasone<br>Hydrocortisone<br>Methylprednisolone<br>Paramethasone<br>Prednisolone<br>Prednisone<br>Triamcinolone	Used as part of the generic name text, stand alone or combination drug

[a] Milligrams of morphine having equivalent strength to 1 mg of indicated substance. Per VA Department of Defense Guidelines for Managing Opioids for Chronic Pain (http://www.healthquality.va.gov/guidelines/Pain/cot/).

©2019 Jasuja GK et al. *JAMA Network Open.*

eTable 2. ICD-9-CM Diagnosis and Procedure Codes

Condition	Names/codes	Rule
**HIV**	042.x-044.x, 079.53, V08	Exclusion Criteria
**Gender Identity Disorder**	302.85	Exclusion Criteria
**Testicular Disease**	186.x, 222.0, 236.4, 604.0, 604.9x, 752.51, 752.52, 758.6, 758.7, V45.77, *procedure codes* 62.3, 62.4x	Exclusion Criteria
**Pituitary Disease**	194.3, 227.3, 237.0, 253.1, 253.7, 275.01, 275.02, 275.03, 377.51, *procedure codes* 07.6x	Exclusion Criteria
**Hypothalamic Disease**	253.2, 253.3, 253.4, 253.5	Exclusion Criteria
**Cancer other than Prostate**	140.x-165.x, 170.x -172.x, 174.x, 176.x, 179, 180.x-184.x, 186.x-208.x, 209.1, 209.2, 209.3, 239.x	Any mention during the previous 12 mo.
**Prostate Cancer**	185	Any mention during the previous 12 mo.
**Pain Indication Category**	Low Back Pain<br>722.xx, 724.xx, 846.xx, 847.xx	Any mention during the previous 12 mo.
	Osteoarthritis<br>715.xx	Any mention during the previous 12 mo..
**Chronic pain conditions**	338.0, 338.2x-338.4, 780.96	Any mention during the previous 12 mo.
**CHF**	398.91, 402.01, 402.11, 402.91, 404.01, 404.03, 404.11, 404.13, 404.91, 404.93, 425.x, 428.x	Any mention during the previous 12 mo.
**COPD**	490, 491.x-493.x, 494, 496, 500-505, 506.4	Any mention during the previous 12 mo.
**Coronary Artery Disease**	410.x-414.x, 429.2	Any mention during the previous 12 mo.
**Diabetes**	250.x, 357.2, 362.0, 366.41	Any mention during the previous 12 mo.
**Hyperlipidemia**	272.0-272.4	Any mention during the previous 12 mo.
**Hypertension**	401.x-405.x	Any mention during the previous 12 mo.
**Kidney Disease, Chronic**	403.x, 405.01, 405.11, 405.91, 581.x-583.x, 585-587, 588.8, 588.9	Any mention during the previous 12 mo.
**Liver Disease, Chronic**	456.0, 456.1, 456.2, 567.23, 572.2, 572.3, 572.4, 572.8, 571.2, 571.4, 571.5, 571.6, 571.8, 571.9, 789.5, V42.7	Any mention during the previous 12 mo.
**Obstructive Sleep Apnea**	327.23	Any mention during the previous 12 mo.
**Peripheral Artery Disease**	440.x-442.x, 443.89, 443.9, V43.4	Any mention during the previous 12 mo.

(continued)

©2019 Jasuja GK et al. *JAMA Network Open.*

eTable 2. ICD-9-CM Diagnosis and Procedure Codes *(continued)*

Condition	Names/codes	Rule
**Stroke**	433.01, 433.11, 433.21, 433.31, 433.81, 433.91, 434.01, 434.11, 434.91, 436, 438.x	Any mention during the previous 12 mo.
**TIA**	435.x	Any mention during the previous 12 mo.
**Alcohol Abuse**	291.x, 303.x, 305.0, 357.5, 425.5, 535.3, 571.0, 571.1, 571.2, 571.3, 790.3, 980, E86.0, V11.3	Any mention during the previous 12 mo.
**Anxiety**	300.00, 300.01, 300.02, 300.09, 300.10, 300.20, 300.21, 300.22, 300.23, 300.29	Any mention during the previous 12 mo.
**Bipolar Disorder**	296.0, 296.1, 296.4, 296.5, 296.6, 296.7, 296.8	Any mention during the previous 12 mo.
**Dementia**	290.0, 29010, 290.40, 290.41, 290.42, 290.43, 294.10, 294.11, 331.0, 331.19, 331.2, 331.7, 331.82, 331.83, 331.89, 331.9, 797	Any mention during the previous 12 mo.
**Depression, Chronic**	296.2, 296.3, 296.82, 300.4, 311	Any mention during the previous 12 mo.
**Other Psychotic Disorders**	297.1, 297.3, 298.0, 298.1, 298.4, 298.8, 298.9	Any mention during the previous 12 mo.
**PTSD**	309.81	Any mention during the previous 12 mo.
**Schizophrenia**	295.x	Any mention during the previous 12 mo.
**Substance Abuse, Non-Alcohol**	292.x, 304.2, 304.4, 304.5, 304.6, 304.7, 304.8, 304.9, 305.3, 305.4, 305.5, 305.6, 305.7, 305.9	Any mention during the previous 12 mo.
Outcomes		
**Major Adverse Cardiovascular Events (MACE)**<br>*Note:In the analysis, a MACE event was considered to be the earliest occurrence of death, myocardial infarction or stroke.*	Myocardial infarction<br>410, 410.xx	Except 410.x2 in inpatient principal discharge field
	Stroke<br>- ICD-9-CM code of 430-432, 434, 436 in the inpatient discharge diagnosis field, OR<br>- ICD-9-CM code of 342, 433, 438 in the inpatient discharge diagnosis field, plus a subsequent ICD-9 code of V57 in inpatient or outpatient record within a year, OR<br>- ICD-9-CM code of 433 in the inpatient discharge diagnosis field, plus two or more subsequent ICD-9 codes of 342 in inpatient or outpatient data within a year.<br>Use admission date of first inpatient stay with code as event date.	
**Bone fractures: Vertebral**	805.xx, 806.xx<br>Clean period: absent at baseline and 12 months prior to the index date	Inpatient or outpatient data (all fields)
**Bone fractures: Femoral or Hip**	808.xx, 820.xx, 821.xx<br>Clean period: absent at baseline and 12 months prior to the index date	Inpatient or outpatient data (all fields)
**Anemia**	hemoglobin A1c<12 g/dL or hematocrit <36%	Lab measurements closest to index date

©2019 Jasuja GK et al. *JAMA Network Open.*

eTable 3. Subgroup Analysis of All-Cause Mortality, Major Adverse Cardiovascular Event, Bone Fractures, and Anemia Outcomes in the One-Year and the Six-Year Follow-up Time as a Function of Long-term Opioid/Testosterone Use Status: Patients Not on Glucocorticoids

	**Unadjusted Estimates**			**Model 1**	**Model 2**
	Outcome Events	Bivariate Hazard Ratio (HR)		Covariate-Adjusted Cox model [a]	PS[b] Matched Cox model
**Outcome**	**N (Unadjusted incidence rate per 100 person-year)**	**HR (95% CI)**		**HR (95% CI)**	**HR (95% CI)**
		**N= 15,149**		**N= 15,149**	**N= 9,468**
**All-cause mortality**					
No-testosterone	119 (1.2)	1.00		1.00	1.00
Testosterone	213 (0.6)	0.46 (0.36-0.57)		0.56 (0.44-0.71)	0.56 (0.43-0.73)
**Incidence of major adverse cardiovascular events [c] or death**					
No-testosterone	227 (2.19)	1.00		1.00	1.00
Testosterone	390 (1.08)	0.48 (0.41-0.57)		0.57 (0.48-0.68)	0.54 (0.44-0.66)
**Incidence of bone fractures (Vertebral fractures. ICD-9 805, 806)**					
No-testosterone	44 (0.43)	1.00		1.00	1.00
Testosterone	95 (0.27)	0.67 (0.47-0.97)		0.66 (0.45-0.96)	0.70 (0.46-1.08)
**Incidence of bone fractures (Femoral or Hip fractures. ICD-9 808, 820, 821)**					
No-testosterone	28 (0.28)	1.00		1.00	1.00
Testosterone	59 (0.17)	0.67 (0.42-1.05)		0.83 (0.51-1.34)	0.69 (0.41-1.17)
**Incidence of bone fractures (Vertebral, Femoral or Hip fractures. ICD-9 805, 806, 808, 820, 821)**					
No-testosterone	67 (0.66)	1.00		1.00	1.00
Testosterone	151 (0.42)	0.71 (0.53-0.95)		0.75 (0.55-1.01)	0.71 (0.50-1.00)
**Anemia**					
Subgroup 1: Patients with baseline anemia [d]					
**Baseline anemia resolved**		**N= 1,005**		**N= 1,005**	**N= 562**
No-testosterone	286 (105.7)	1.00		1.00	1.00
Testosterone	549 (136.3)	1.30 (1.12-1.50)		1.29 (1.01-1.51)	1.18 (0.98-1.42)
Subgroup 2: Patients without baseline anemia [d]					
**New anemia emerged**		**N= 12,305**		**N= 12,305**	**N= 7,522**
No-testosterone	730 (11.9)	1.00		1.00	1.00
Testosterone	1,440 (6.9)	0.62 (0.56-0.68)		0.71 (0.64-0.78)	0.70 (0.63-0.78)

a) Adjusted for age, gender, race, marital status, BMI, copay requirement, zip code poverty level, and baseline status of the following clinical conditions: indications for pain, chronic pain conditions, congestive heart failure, cancers, coronary artery disease, hypertension, diabetes, hyperlipidemia, liver disease, chronic kidney disease, stroke/transient ischemic attack, dementia, depression, bipolar disease, substance use disorder, alcohol dependence, psychosis and use of antipsychotic medications; b) Propensity score; c) Incident cases (new occurrence) of myocardial infarction or thrombotic stroke or death (appendix 2); d) Based on measurements that were closest to the index date. Anemia is defined as hemoglobin<12 g/dl or hematocrit<36%; Included patients had to have both pre and post index hemoglobin/hematocrit values.

©2019 Jasuja GK et al. *JAMA Network Open.*

eTable 4. Analyses of Subsets of Testosterone Users Using a Specific Testosterone Formulation in Comparison With All Non-Testosterone Users

	**Unadjusted Estimates**			**Model 1**
	Outcome Events	Bivariate Hazard Ratio (HR)		Covariate-Adjusted Cox model [a]
**Outcome**	**N (Unadjusted incidence rate per 100 person-year)**	**HR (95% CI)**		**HR (95% CI)**
**All-cause mortality**				
**All formulations** [b]		**N= 21,272** [b]		**N= 21,272** [b]
No-testosterone	203 (1.4)	1.00		1.00
Testosterone	327 (0.7)	0.41 (0.34-0.49)		0.51 (0.42-0.61)
**Injection only**		**N= 16,011**		**N= 16,011**
No-testosterone	203 (1.4)	1.00		1.00
Testosterone	245 (0.7)	0.45 (0.37-0.54)		0.56 (0.46-0.68)
**Gel only**		**N= 9,206**		**N= 9,206**
No-testosterone	203 (1.4)	1.00		1.00
Testosterone	31 (0.5)	0.32 (0.22-0.47)		0.40 (0.27-0.59)
**Patch only**		**N= 10,290**		**N= 10,290**
No-testosterone	203 (1.4)	1.00		1.00
Testosterone	50 (0.5)	0.32 (0.23-0.43)		0.39 (0.28-0.53)
**Incidence of major adverse cardiovascular events** [c] **or death**				
**All formulations** [b]		**N= 21,272** [b]		**N= 21,272** [b]
No-testosterone	358 (2.5)	1.00		1.00
Testosterone	605 (1.2)	0.48 (0.42-0.54)		0.58 (0.51-0.67)
**Injection only**		**N= 16,011**		**N= 16,011**
No-testosterone	358 (2.5)	1.00		1.00
Testosterone	442 (1.3)	0.52 (0.45-0.60)		0.64 (0.55-0.74)
**Gel only**		**N= 9,206**		**N= 9,206**
No-testosterone	358 (2.5)	1.00		1.00
Testosterone	58 (0.9)	0.36 (0.27-0.47)		0.44 (0.33-0.58)
**Patch only**		**N= 10,290**		**N= 10,290**
No-testosterone	358 (2.5)	1.00		1.00
Testosterone	102 (1.0)	0.39 (0.31-0.49)		0.46 (0.36-0.57)

a) Adjusted for age, race, marital status, BMI, copay requirement, zip code poverty level, and baseline status of the following clinical conditions: indications for pain, chronic pain conditions, use of glucocorticoids, congestive heart failure, cancers, coronary artery disease, hypertension, diabetes, hyperlipidemia, liver disease, chronic kidney disease, stroke/transient ischemic attack, dementia, depression, bipolar disease, substance use disorder, alcohol dependence, psychosis and use of antipsychotic medications; b) Includes 67 cases where mode of administration was other; c) Incident cases (new occurrence) of myocardial infarction or thrombotic stroke or death (eTable 2).

©2019 Jasuja GK et al. *JAMA Network Open.*

eTable 5. Correlation of a Potentially Unobserved Confounder With All-Cause Mortality and Long-term Opioids With Testosterone

Correlation of Unobserved Confounder[a] with All-Cause Mortality	Correlation of Unobserved Confounder[a] with Long-term Opioids andTestosterone	Hazard Ratio for Death (95% CI); p-value
-0.15	0.10	0.95 (0.75-1.14), p=0.56
-0.10	0.15	1.04 (0.87-1.25), p=0.66
-0.08	0.20	1.03 (0.86-1.24), p=0.74
-0.06	0.25	0.96 (0.80-1.16), p=0.69
-0.06	0.30	1.09 (0.90-1.32), p=0.37
-0.05	0.35	0.96 (0.79-1.16), p=0.65

[a] Confounder assumed to have prevalence of 0.50 in analytic population.

©2019 Jasuja GK et al. *JAMA Network Open.*
